# Ossification of the Ligamentum Flavum of the Lumbar Spine

**DOI:** 10.7759/cureus.19023

**Published:** 2021-10-25

**Authors:** Talles Sidronio, Sanjeev Kumar

**Affiliations:** 1 Anesthesiology, University of Florida College of Medicine, Gainesville, USA

**Keywords:** decompression, laminectomy, chronic pain, spinal stenosis, ligamentum flavum

## Abstract

Ossification of the ligamenta flava (OLF) is a rare and likely multifactorial condition that most commonly affects the lower thoracic spine. It can result in chronic pain and neurological deficits and has a higher prevalence in men of Japanese descent. We present the case of OLF of the lumbar spine in a Caucasian man with a history of multiple sports-related injuries. To treat his severe lumbar spinal stenosis, the patient underwent endoscopic intralaminar laminotomy with partial facetectomy and complete resection of OLF with lateral recess decompression, which immediately resolved his symptoms. We noted signs of OLF based on T1 and T2 signal changes upon re-evaluation of MRI.

## Introduction

Ossification of the ligamenta flava (OLF) occurs as a result of elastic fiber breakdown and subsequent chondrometaplasia and endochondral ossification of the ligament [1,2]. The etiology of OLF is likely multifactorial, with a higher prevalence in men of Japanese descent [1,3]. The available literature does not provide potential reasons or theories for the higher prevalence in Japanese men. At the molecular level, the type II collagen that is more characteristic of cartilage is replaced by type I collagen, which is responsible for 90% of the extracellular matrix in bone architecture [4]. This change is largely driven by mechanical stress, which upregulates a variety of signaling pathways responsible for osteogenic differentiation [4,5].

The pathology is largely inconsequential and more often discovered as an incidental finding on routine imaging. In the Japanese population, for example, OLF is detected in 4.5% of outpatient X-rays [4]. However, stenosis of the spinal canal and neuroforamina may cause nerve impingement, and, subsequently, the development of chronic pain and neurologic compromise. The most commonly affected region is the lower thoracic spine, followed by the upper and middle thoracic spine, the cervical spine, and least commonly the lumbar spine [1,3]. We present the case of OLF of the lumbar spine in a Caucasian man with a history of multiple sports-related injuries. Multiple trauma or injuries to the spine has not been described as an independent risk factor for OLF in the literature but being a rare disease it might be that this association has not been studied yet.

## Case presentation

This case describes a 65-year-old Caucasian man who presented to our clinic with a six-year history of severe chronic lumbago with neurogenic claudication leading to the inability to walk more than a few steps. His past medical history included anxiety, atrial fibrillation after past ablation, coronary artery disease after percutaneous coronary angioplasty with three stents, gastroesophageal reflux disease, hypertension, obstructive sleep apnea, and hyperlipidemia. His pain was mainly axial and involved the lumbar spine with radiation to bilateral anterolateral thighs. He endorsed a history of over 100 falls during his years as an ice skater but described the onset of his pain as gradual. The patient had no history of any of the reported risk factors of OLF in literature, like nephrolithiasis, hypercalcemia, or kidney disease and his laboratory values for serum calcium, creatinine, and glomerular filtration rate were within normal limits. Prior interventions included physical therapy, acetaminophen, nonsteroidal anti-inflammatory drugs, pregabalin, and multiple caudal epidural steroid injections over the years with only marginal benefit. MRI revealed the etiology of his lumbar radiculopathy to be severe lumbar spinal stenosis.

**Figure 1:**
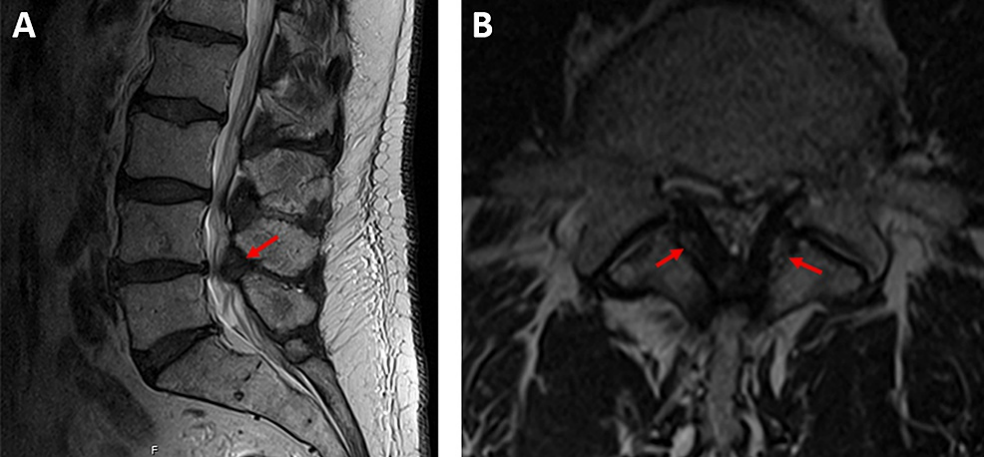
T2-weighted magnetic resonance images (A) Mid-sagittal view depicting severe L4-5 stenosis and ossification of the ligamentum flavum (red arrows). (B) Transverse view depicting severe L4-5 stenosis and ossification of the ligamentum flavum (red arrows).

**Figure 2:**
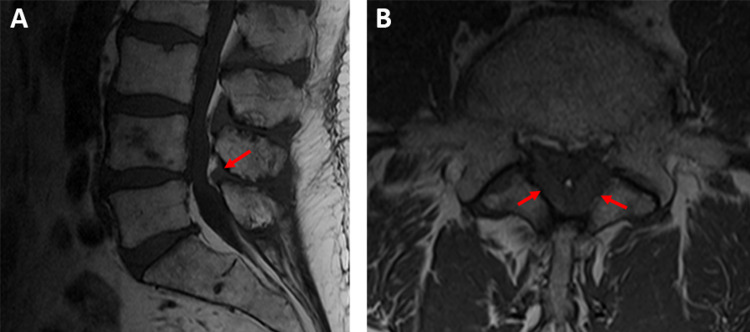
T1-weighted magnetic resonance images (A) Mid-sagittal view depicting severe L4-5 stenosis and ossification of the ligamentum flavum (red arrows). (B) Transverse view depicting severe L4-5 stenosis and ossification of the ligamentum flavum (red arrows).

On examination, the patient was sitting. He could only stand for a few seconds upright before leaning forward and taking just a few steps before he had to sit due to cramping in both legs attributed to neurogenic claudication. He exhibited 5/5 strength in his bilateral lower extremities with no signs of muscle wasting or atrophy. His deep tendon reflexes were 2/4 in the bilateral lower extremities. The facet loading test was positive bilaterally in the lumbar region. MRI of the spine revealed central canal narrowing and bilateral neuroforaminal narrowing from L3-5. At L4-5, there was a broad-based posterior disc bulge with annular fissuring of the nucleus pulposis and mild facet arthropathy. In retrospect, ligamentum flavum ossification was suggested with T2- and T1-weighted images (Figures 1-2).

The patient underwent endoscopic intralaminar laminotomy with partial facetectomy and lateral recess decompression with immediate resolution of his symptoms. Intraoperatively, we identified OLF at the L4-5 that had not previously been noted on MRI.

**Figure 3:**
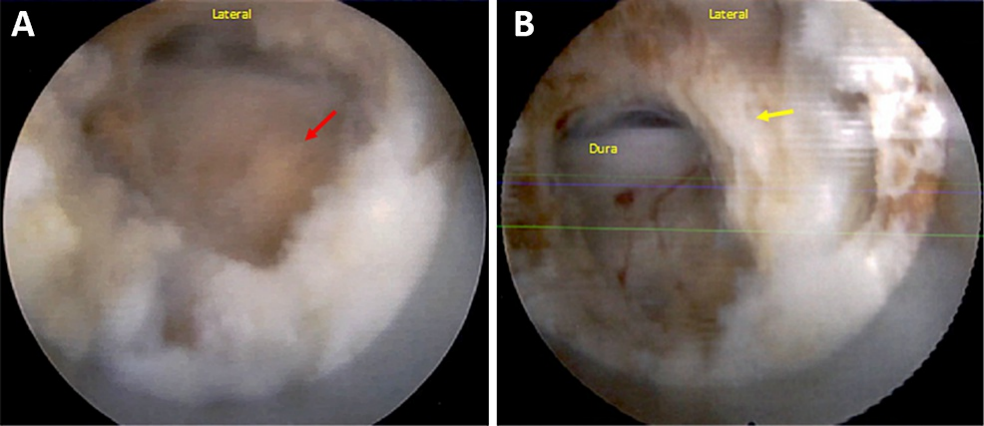
Endoscopic images of the dorsal fibers of the ligamentum flavum (A) and the ossified ventral fibers (B)

The patient underwent uneventful induction of general anesthesia and neuromonitoring was performed via the quadriceps, anterior tibial, gastrocnemius, and hamstring on the right side. A true anteroposterior view of the lumbar spine at the L4-5 level was obtained by using fluoroscopy. A working channel was established and the spinal endoscope was introduced. The descending lamina was identified in the right L4-5 interlaminar window and a laminotomy was created over the right-sided descending lamina down to the descending facet. Once at the ligamentum flavum, the dorsal fibers were taken down, revealing ventral fibers of the ligamentum flavum that were unexpectedly found to be hardened and ossified across the interlaminar window at L4-5 (Figure 3). The hardened ossified shell of the ligamentum flavum was carefully dissected. The dura was fully decompressed and freely mobile, without signs of ossification. Once adequate hemostasis was visualized, 40 mg of methylprednisolone was injected at the site and the incision was closed.

The patient reported immediate improvement of his radicular symptoms with no neurological deficits in the postanesthesia care unit. The patient presented to the clinic for a follow-up on postoperative day 9 with sustained improvement and was neurologically intact.

## Discussion

The ligamenta flava is a series of ligamentous structures that connect the ventral laminae of adjacent vertebrae from C2 to S1 [6]. They derive their name from their yellow coloring, which is the result of a high elastin content. Over time, a decrease in elastin is seen and the ligaments may undergo hypertrophy, calcification, or ossification. These changes, and ossification, in particular, can lead to myelopathy and radiculopathy [7]. It has been documented that the incidence of OLF is higher in patients with hypercalcemia, diabetes mellitus, ankylosing spondylitis, spinal hypermobility, and diffuse idiopathic skeletal hyperostosis [1,2,8].

Furthermore, Li et al. [8] reported electron microscopy findings suggestive of common features among patients with thoracic OLF: elastic fiber breakdown, proliferation of collagenous fibers, calcium crystal deposits, chondrometaplasia, and endochondral ossification. Although the etiology of these ultrastructural changes is multifactorial, most patients in that study worked as heavy laborers and mechanical stress was likely a major contributor.

Decompressive surgery is the definitive treatment in symptomatic patients and typically leads to favorable outcomes [8]. Interestingly, there have been reports of OLF recurring at the same intervertebral level of previous OLF decompressions [9]. Roughly 20% of patients with OLF have multiple segments of involvement, which may complicate surgical planning. Lee et al. have proposed a method by which OLF-induced myelopathy can be diagnosed via MRI with 87.1% sensitivity and 87.3% specificity [10]. This might be of particular use in instances in which the OLF burden spans more than one intervertebral segment or in patients with atypical clinical presentation. Routine X-ray may detect OLF; however, tissue characterization and degree of neurologic compression are better elucidated with a combination of MRI and CT [11].

## Conclusions

In our case, upon re-evaluation of MRI, we noted in retrospect signs of OLF based on T1 and T2 signal changes. In retrospect, OLF could have been further elucidated with a preoperative CT scan. We encourage clinicians to consider CT imaging if there is suspicion of OLF on MRI to facilitate better informed preoperative surgical planning. For this patient, endoscopic intralaminar laminotomy with partial facetectomy and complete OLF resection with lateral recess decompression resulted in immediate improvement in radicular symptoms with no neurological deficits.
